# Positive affect and heart rate variability: a dynamic analysis

**DOI:** 10.1038/s41598-024-57279-5

**Published:** 2024-03-25

**Authors:** Tony Beatton, Ho Fai Chan, Uwe Dulleck, Andrea Ristl, Markus Schaffner, Benno Torgler

**Affiliations:** 1 School of Economics and Finance, Queensland University of Technology, 2 George St, Brisbane, QLD 4000 Australia; 2 Centre for Behavioural Economics, Society and Technology (BEST), 2 George St, Brisbane, QLD 4000 Australia; 3 Crawford School of Public Policy, Australian National University, Canberra, Australia; 4 Center for Economic Studies, CESifo Ludwig-Maximilians-Universität, Munich, Germany; 5 Heart2Business GmbH, 1070 Vienna, Austria; 6 CREMA-Center for Research in Economics, Management and the Arts, Südstrasse 11, 8008 Zürich, Switzerland; 7 Australian Research Council Training Centre for Behavioural Insights for Technology Adoption (BITA), Queensland 4000 Brisbane, Australia; 8 University of Canberra, Canberra, Australia

**Keywords:** Positive affect, Heart rate variability, Data collection, Analytical models, Interpretation, Psychology, Human behaviour

## Abstract

Traditional survey methods can provide noisy data arising from recall, memory and other biases. Technological advances (particularly in neuroscience) are opening new ways of monitoring physiological processes through non-intrusive means. Such dense continuous data provide new and fruitful avenues for complementing self-reported data with a better understanding of human dynamics and human interactions. In this study, we use a survey to collect positive affect (feelings) data from more than 300 individuals over a period of 24 h, and at the same time, map their core activities (5000 recorded activities in total) with measurements of their heart rate variability (HRV). Our results indicate a robust correlation between the HRV measurements and self-reported affect. By drawing on the neuroscience and wellbeing literature we show that dynamic HRV results are what we expect for positive affect, particularly when performing activities like sleep, travel, work, exercise and eating. This research provides new insights into how to collect HRV data, model and interpret it.

## Introduction

Surveys are a key tool for almost all fields that explore human nature, with self-reporting as the most common means of measuring emotional experience. Thus, analyzing data on individuals’ subjective judgment is a dominant empirical strategy in social science; often, there is simply no other method available to collect such data. Herein lies a key problem with the key tool: good science depends on unbiased knowledge from which to derive conclusions, yet subjective self-reports are subject to a number of confounding factors, particularly social desirability effects. Hence, recent questions in the subjective well-being literature about the reliability of survey answers around measures of experience, mood, and feelings are well justified^1–5^, especially as policymakers are becoming increasingly interested in the value of subjective well-being as a policy tool^6–9^. Subjective measures in general are difficult to quantify, interpret, and categorize^10^.

However, new research opportunities have evolved thanks to significant technological advances, including the use of non-intrusive wearable sensors that allow mapping the behaviors and interactions of individuals in the real world. Such technologies can provide a dynamic digital footprint or digital breadcrumbs of our society. Some scholars have even referred to such technologies as “social fMRI’s”^11^. Medical research, for example, also refers to such real-time analysis as adaptive or interactive monitoring^12^. It is therefore a natural avenue to explore whether results obtained from these ‘reality mining’ instruments correlate with important societal measures such as subjective well-being. In this study, we attempt to identify such a correlation to determine whether high-frequency data can complement the use of survey data, thereby opening up access to continuous behavioral data. Confirming this complementarity also offers the opportunity to deal with inherent problems of survey data such as reporting biases, memory errors, and other potential biases^13–15^; although, the literature on subjective well-being has tried to overcome such biases by applying approaches such as the day reconstruction method (DRM)^16^ rather than relying on annually collected data.

## Review of related literature

Using high frequency data also offers the opportunity for more detailed exploration of environmental and situational aspects. One advantage of non-intrusive instruments is that individuals forget they are being measured^17^, which means that they are also able to wear the instruments throughout an entire day while doing all their daily activities. This offers a valuable opportunity to shed more light on how daily single major activities matter. Thus, in our study we follow 300 individuals over a period of 24 h, mapping a total of 5000 core daily activities and combining those activities with the measurements of their heart rate variability (HRV) and their assessment of how they feel (positive affect). As such, we measure the momentary aspect of feelings closer to the activity or daily life experience than does the DRM approach, which relies on respondents to revive their memories of the previous day^16^. This means that we rely more heavily on ecological momentary assessment, which reduces errors and biases associated with retrospection such as recall biases or heuristic biases or strategies in general^18^. However, both measures tend to cover hedonic happiness (more momentary feeling of pleasure sensation) rather than eudaemonic happiness, which focuses more on the lasting element or feeling. We will therefore refer to positive affect during our empirical analysis.

Linking physiological and emotional responses to our daily activities provides more insights into experienced utility rather than just the decision utility that has been the core focus of economists^19^. Fields such as clinical psychology, health psychology, or behavioral medicine, for example, have focused on the study of daily life as a way of identifying the bright and the dark side of human behavior, emotions, and experiences in the form of pain or pleasure during their activities^20,21^; for example, by using daily diaries to explore how specific factors (such as food consumption) affect well-being^22^.

Activities, routines, and rituals across the day can affect how we feel. For example, closeness to our family and friends is associated with feeling happier^23^, especially in children^24^, and enjoyable daily activities can boost happiness in older persons^25^. Predictability in family routine and daily activities improves the happiness of children^26^, and adolescents^27^, especially if they have a disability such as autism^28^. Using a survey questionnaire, Darviri et al.^29^ showed that personal choices like how and when we sleep, dietary choices, physical exercise, and the social and mental balance in our daily routine can affect the stress in our lives and our health and happiness. Daily activities like commuting^30^, working^31^, when we eat^32^, religious participation, and exercise affect our happiness^33^. Using two subjective data sources, socio-economic survey, and DRM data, Möwisch et al.^34^ showed that daily activities affect our happiness. In our study we try to go one step further by also employing objective heart rate monitoring data to gauge how we feel. While survey-based research has studied how the flow of our daily experiences^35^ and the situational context^36^ of our daily activities affects our feelings, we are not aware of research that has compared and contrasted subjective survey data, DRM comparable data, as well as objective heart rate monitoring data into examining the importance of daily activities (including the order of daily activities) to better understand happiness, or more particularly how we feel why going about our daily activities. Like happiness, affect is dynamic and relative; for a discussion on happiness/well-being see^1–4,7,37,38^.

## Materials and methods

### Activity log and mood assessment

Upon signing up for the study, participants were asked to fill in an activity protocol (Appendix, Fig. S1), in which they report the type of the current activity conducted (e.g., communication, eating, transport, sleeping) and their current mood during the activity, over the 24-h period (Appendix, Fig. S2). The start time and end times (in hour-minute) of each activity were also reported at the beginning and termination of the activity and were then used to align with the measurement from the heart rate monitor. The protocol required participants to make a choice between either positive or negative feelings, with three values on each valency ranging from very poor to excellent (Appendix, Fig. S1), enabling us to measure the intensity of emotional affect during each daily activity. Since the distribution of the self-reported measurement of mood is heavily skewed towards the positive, we merged the lowest two categories into one to create our measure of positive affect (Appendix, Fig. S3). The distribution of positive affect during different activities is summarized in the supplementary online material (Appendix, Fig. S4).

Given the continuity of the protocol, the data allows direct access to the individuals’ self-reported well-being without major time delays, thereby providing a proxy for experiential knowledge. Eliminating the delay between experience and its report lessens information loss from retrieving such information at a later stage, which would be subject to a retrospective bias^39^. The simplicity of the protocol in collecting minimal information reduces the burden and facilitates prompt recording of their emotions. Sampling methods that attempt to measure experience and feelings in real time have been criticized for being impractical for large samples^16^, especially given the cost, which is often prohibitive^40,41^.

Another major advantage of the protocol is that, rather than simply posing a single question in repeated settings^42^, it assesses momentary experience for each single episode of an activity throughout the day. As discussed beforehand, such measurement is closely related to the concept of happiness, which manifests in physiology and is linked to both positive and negative affect and current mood state^2^. As these 24-h measurements were taken during normal daily conditions, the current design is well suited to exploring human nature as the normal day-to-day activities captured are likely representative of real-life environments^42^.

### Heart rate variability (HRV) measure

At the beginning of the observation period, each participant was fitted with a noninvasive pocket-sized heart rate monitor (Appendix, Fig. S5) that records an electrocardiogram (ECG), thereby allowing nonintrusive exploration of the relations between natural variations in the participants’ physiological activity and psychological states. Specifically, by examining the HRV, the excitatory sympathetic (fight and flight) and parasympathetic (rest and relax) activity in the autonomic nervous system can be identified and analysed^43^. The sympathetic system (SNS) affects the heart rate through the sympathetic nerves by releasing cell-stimulating hormones (e.g., epinephrine and norepinephrine) into the bloodstream, with the efferent sympathetic nerves innervate the heart, causing a slower but longer lasting (than the vagal system) influence on heart rate. The parasympathetic system (PNS) is responsible for rest and relaxation, like SNS, it influences heart rate by interacting with the heart’s intrinsic cardiac nervous system (ICNS). The vagus nerve, a key component of the parasympathetic system, connects to the ICNS, which then modulates the activity of the sinoatrial (SA) node, the heart’s primary pacemaker, to decrease heart rate (mediated by the neurotransmitter acetylcholine)^44^. The extent of sympathetic and parasympathetic activity can be identified based on heart rate variations, which occur at different speeds or frequencies^45^. Those reflecting activity by the sympathetic system are considerably longer (maximum effect after more than 5 s = low frequency changes) than those reflecting activity by the parasympathetic system (maximum effect after less than 5 s = high frequency changes)^46^.

Because either an increase in sympathetic cardiac control, a decrease in parasympathetic control, or both^47^ tends to be associated with stressors, HRV analysis with ECG data is often used to identify psychological, emotional, and mental activities^45,48–53^. We employ the standard Fast Fourier transformation (FFT) to calculate the ratio of activity in the low frequency band to activity in the high frequency band (i.e., the LF/HF ratio). This ratio (mean = 2.14, SD = 0.364) offers a more specific and efficient measure for autonomic regulation (to differentiate between the activity of the SNS and PNS) than other time-domain methods (e.g., Mean HR, RMSSD, PNN50, or SDNN)^44^ and provides an index of sympathovagal balance^45^, which serves as a useful indicator of psychological strain^54^ (for the value distribution, see Appendix, Fig. S6). The LF/HF ratio tends to increase in response to heightened stress, which is typically linked to elevated SNS activity, and characterized by a rise in the low-frequency (LF) band and a decrease in overall HRV^44^. By using repeated observations over an active day, we can control for the variation of biological indicators over time.

However, it is crucial to clarify that a higher LF/HF ratio does not necessarily indicate change overall HRV. In fact, it could have various relationships with HRV, particularly when accompanied by an increase in heart rate due to cycle length dependence effects^55^. Moreover, it is recognized that the relative sympathovagal balance continually adjusts to meet regulatory system demands^56^. For example, in ambulatory recordings like those used in our study, LF power can be augmented by short-term sympathetic activations, which may cause lower frequency rhythms to overlap into the LF band. Additionally, in situations where there is prolonged sympathetic activity (often due to physical activity), shifts in the sympathetic and parasympathetic balance are more accurately indicated by increases in HR. Thus, to provide a more robust understanding of these dynamics, we employed two key analytical approaches (also see Extended Validation section in the Online Supplementary Material).

Firstly, we control for heart rate (HR) in the regression analysis. This approach allows us to account for the potential cycle length dependence effect of changes in HR on the HRV through shift in the balance of autonomic nervous system activity, especially during physical activity or periods of prolonged sympathetic activation. By adjusting for HR, we can provide a more accurate interpretation of the LF/HF ratio, considering the dynamic nature of the autonomic nervous system regulation.

Secondly, we explored the relationship between the LF/HF ratio and HR and RMSSD (Fig. S12c and S12d), a metric that more directly reflects HRV (primarily influenced by parasympathetic activity). Additionally, we extended our analysis to investigate the impact of changes in HRV (as measured by RMSSD) on positive affect (see Table S6). The results from these analyses reinforce our main findings involving the LF/HF ratio.

Finally, to further discern whether changes in the LF/HF ratio are the result of either increased sympathetic activity (reflected in higher LF power), reduced parasympathetic activity (lower HF power), or combination of both, we conducted a systematic regression analysis to understand the influences of LF and HF on the LF/HF ratio (Fig. S12a). Our findings indicated that, while both HF and LF influence LF/HF ratio, HF has a relatively larger impact than LF. Further, individual-specific analyses further confirmed that the magnitude of HF’s influence exceeds that of LF (Fig. S12b).

### Ethics statement

This research project has been conducted in accordance with the National Statement on Ethical Conduct in Human Research (QUT Institutional Review Board Project ID 5699). Participants have been fully informed about the nature and purpose of the research, and their voluntary participation has been obtained through informed consent.

## Sample

The data collection took place between January 9, 2006, and August 21, 2008. The participants were recruited through medical practitioners (GPs and gynaecologists) and firm-specific contacts who took part in a 24-h heart rate variability measurement program as part of a lifestyle appraisal. In total 344 Austrian residents (mostly Viennese) were asked to fill out the mood/well-being assessment part of the activity protocol. Information obtained from the activity protocol was manually coded and entered into machine-readable format. Customized MATLAB scripts were developed to extract and calculate related HRV measures from individual’s ECG records and combine with activity logs and mood assessment. Before the analysis, we proceeded with the following data cleaning procedure (sanity checks) to minimize data errors due to measurement and coding entry in the final dataset. First, the data being cubically interpolated to a 5 Hz signal to obtain a continuous measure from the consecutive QRS data points (see^57^). To address abnormalities of ECG data (noise of the recording and misreadings), we dropped observations with the top 1 percentile of noise as well as those with HR in the top and bottom 1 percentile. Then, we removed observations from participants who self-reported their age as younger than 18 or older than 80 (57 participants). Next, to ensure the data have sufficient variation in the types of activities, we removed observations from participants who reported less than 9 h of activity time (the total study duration is on average 21.5 h (SD = 3.9)) (28 participants) and observations where duration was not appropriately recorded (18 participants). Furthermore, when analyzing HRV, we excluded sleeping (*n* = 2269) and other activities with duration less than five minutes (*n* = 145) or longer than 10 h without intermission (*n* = 1143). This leaves the final sample of 1152 participants with 18,693 activities in which 321 participants (5575 activities) completed the mood assessment. Overall, 89% of the participants took part in the 24-h study during weekdays, and 84% began the observation period between 8 am to 6 pm. Participants are, on average, 43.2 years old (± 12.3 SD) (for the age distribution, see Appendix, Fig. S7) with female-to-male ratio of 1:1.49. Participants’ data are anonymized in accordance with ethical requirements. The data were collected by Autonom Talent, an Austrian HR and coaching consultancy using HRV measurements to inform training programs. Participants gave informed consent to participate in the study and agreed to their data been used for further analysis by Autonom Talent and its research partners. Additionally, Autonom Talent published its engagement with the host university on its webpage. Participants were provided with invitations that communicated the research collaboration as well as the intent and results of the study. In addition, participants were informed (in written form and in conversations) that they could withdraw their data from further analysis. The study has been conducted in accordance with the National Statement on Ethical Conduct in Human Research. The experimental protocol and methods were approved by the Queensland University of Technology (QUT) Review Board (Project ID 5699).

## Results

**Figure 1:**
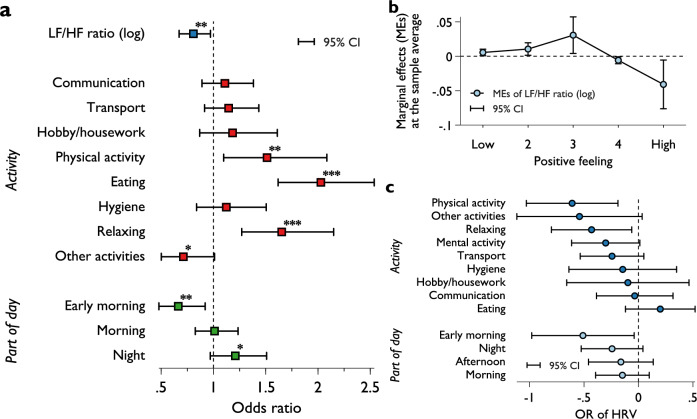
Correlation between positive affect and mental stress. (**A**) Odds ratios (OR) obtained from fixed-effects ordered logit models of positive affect (specification (3) Appendix, Table S1). The reference category for activity and part of day is *mental activity* and *afternoon*, respectively. (**B**) Marginal effects at the sample average of LF/HF ratio on all positive affect outcomes. (**C**) shows the estimated OR for LF/HF ratio for each activity and part of the day, based on models including their interaction terms. *N* = 5414 with 310 participants. Error bars represent 95% CI. *, **, and *** indicate 10%, 5%, and 1% level of significance, respectively.

We use fixed-effects ordered logit models^58,59^ to explore within-subject covariation between psychological state (positive affect) and mental stress (log of LF/HF ratio); see Appendix for the analytical models. We find a negative correlation between mental stress and psychological state. Subjects experiencing higher LF/HF ratio during an activity reported lower positive affect scores (Fig. 1A). For example, a 10 percent increase in LF/HF ratio reduces the odds of reporting of more positive affect (categories above 1) by 1.92% (*p* = 0.024). More specifically, experiencing a 10% raise in LF/HF ratio increases the probability of reporting the lowest 3 affect categories by 0.056 percentage points (pp), 0.1 pp, and 0.31 pp, respectively, and decreases the probability of reporting the 4th and 5th positive affect categories by 0.058 pp and 0.41 pp, correspondingly (Fig. 1B). Such association is highly consistent across various model specifications controlling for different factors; for example, the type of activities conducted, time of the day, and duration of the activities (Appendix, Table S1); employing the BUC-τ estimator^60^ assuming constant thresholds (Appendix, Table S2); or a linear panel fixed-effects model (Appendix, Table S3). The positive-feeling– LF/HF ratio relationship is also consistent for most activities (except for eating, χ^2^(8) = 16.62, *p* = 0.0343) and across the day (χ^2^(4) = 7.78, *p* = 0.0998) (see interaction effects shown in Fig. 1C).

In terms of activities, participants reported more positive feelings during exercise, meals, and relaxing (Fig. 1A), compared to conducting mental activity (work), with odds ratios increasing by about 50%, 100%, and 70%, respectively. Other non-work activities also tend to have a positive effect (without being statistically significant). Interestingly, the meta-analysis by Biskup et al.^31^ found no significant differences in positive affect between work versus non-work domains, pointing out that work is a positive for some people. Overall, we find that the joint effect of non-mental activities is statistically significant (χ^2^(1) = 6.01, p = 0.0142, with an average OR of 1.35). Additionally, a separate regression with non-mental activities grouped together reports a similar result, indicating lower self-reported positive effect during mental activities (OR = 1.29, p = 0.004). Feelings improve throughout the day as participants felt more positive at later times of the day (5 pm–12 am) and more negative during early morning (12 am–6 am), compared to afternoon (12–5 pm) (Fig. 1A, part of the day). We also find that the level of mental stress varies across types of activities conducted and when they are conducted (Appendix, Fig. S8) as we examine their effects on LF/HF ratio using a panel fixed-effects model (Appendix, Table S4).

**Figure 2:**
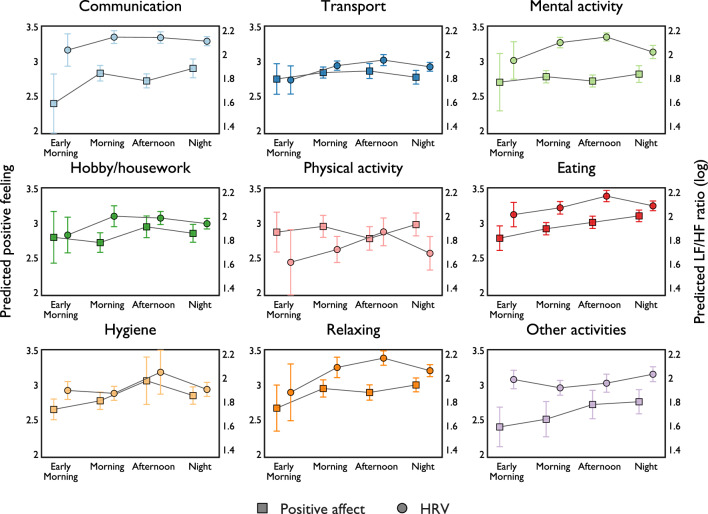
Positive affect and physiological stress level during different activities at various times of day. Predicted levels of positive affect and physiological stress are obtained from fixed-effects models with full interaction terms between activity type and part of day (Appendix, specification 7 Table S3 and specification 6 Table S4, respectively). Error bars represent 95% CI.

Our results indicate that the level of physiological stress is lowest during physical activities, followed by transport, hygiene, and hobby/housework (Appendix, Fig. S8). In contrast to the results on positive affect, we did not find that eating and relaxing have a significant effect on lowering LF/HF ratio (compared to mental activities). In addition, LF/HF ratio is lowest during activities conducted in early morning and highest in the afternoon. A closer look at how different activities conducted during various parts of the day influences affect reveals that individuals feel more positive as the day progresses (Fig. 2, square markers), especially for activities such as *communication*, *eating*, *hobby/housework*, *hygiene*, and *relaxing*. In contrast, positive feeling during *transport*, *mental activity*, and *physical activity* do not seem to vary significantly throughout the day. Interestingly, we observe similar patterns in terms of physiological stress levels for most activities; LF/HF ratio increases as the day progresses and peaks in the afternoon.

**Figure 3:**
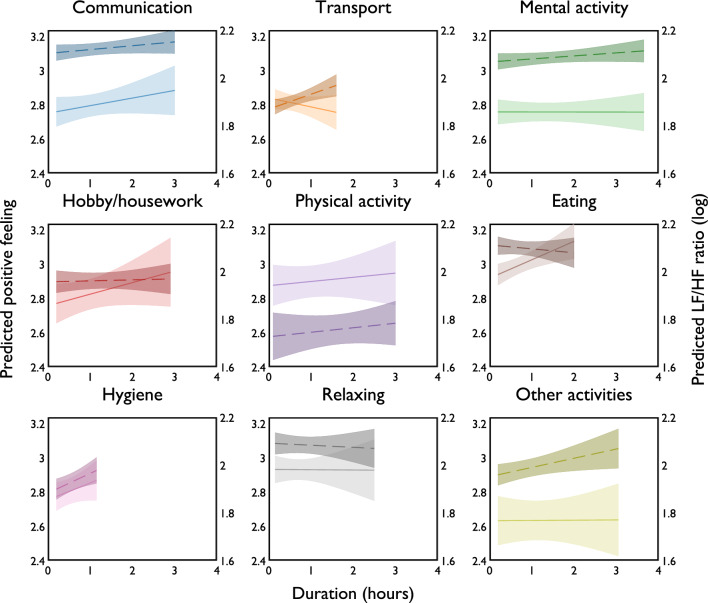
Activity duration on positive feeling and physiological stress level. Predicted levels of positive affect (solid lines) and physiological stress (dashed lines) are obtained from fixed-effects models with interaction terms between activity type and activity duration (Appendix, specification 9 Table S3 and specification 8 Table S4, respectively). Shaded areas represent 95% CI.

We also observe that the positive feeling experienced may depend on duration of the activities (Fig. 3, solid lines). To avoid extrapolation, we show the prediction for duration ranges within the 5th and 95th percentile values of each type of activity. For some activities, such as *eating*, *hygiene*, *communication*, and *hobby/housework*, higher values of positive feeling were reported when the duration of the activity is longer. In contrast, we find no change in positive feeling for long periods of *mental activity* and *relaxing* and a decrease in positive feeling for spending longer time on *transport*. A likely explanation is that participants who enjoy leisure activities chose to increase its duration, while the length of work and travel time are less controllable. In fact, we find that prolonged *transport* and *hygiene* lead to increased physiological stress (elevated LF/HF ratio levels, see Fig. 3, dashed lines).

**Figure 4:**
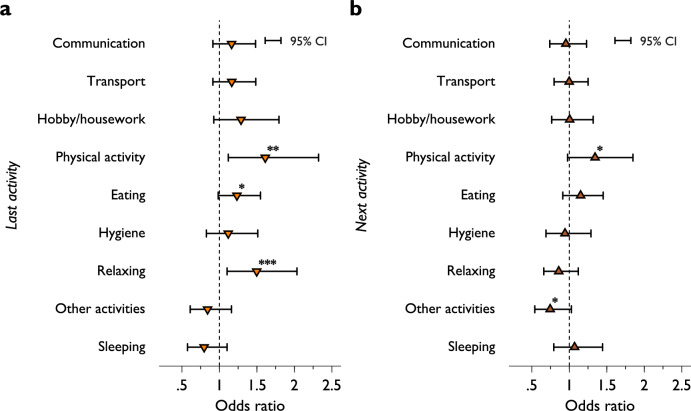
Effect of previous and next activities on positive feeling. ORs obtained from fixed-effects ordered logit models of positive affect including indicators of past (Appendix, **A**, specification (1) Table S5) and next (**B**, specification (2) Table S5) activities. The reference category for activities is *mental activity*. Error bars represent 95% CI. *, **, and *** indicate 10%, 5%, and 1% level of significance, respectively.

A core advantage of our data is the ability to explore how previous activities affect subsequent activities, while also exploring how prospection impacts positive affect. As Railton^61^ (p. 6–7) points out, “[a] prospecting mind must do the “seeing” and “feeling” that simulate what a future will be like, and thereby place future possibilities on all fours with what is actually seen and felt at present”. Such an exploration is difficult when using the DRM approach due to systemic data collection biases. In addition, combining prospection with HRV data can provide further insights; therefore, we examine whether the contemporaneous positive feeling is correlated with previous or future activities. To do so, we include a set of indicators of the preceding (Table S5 specification (1)) and proceeding (Table S5 specification (2)) activity in our regression models, whilst controlling for the type of current activity conducted as well as the current LF/HF ratio. We find that individuals reported higher positive feelings during tasks following *physical activities*, *eating*, or *relaxing* by 1.6, 2.1, and 1.7 times, respectively, compared to the baseline (*mental activity*, Fig. 4A). Self-reported positive feeling is also higher when the individual’s next task was *physical activities* (1.34 times higher) (Fig. 4B). On the other hand, we did not find a significant change in positive feelings before or after *communication, transport*, *hobby/housework*, *hygiene*, or *sleep*, compared to the baseline. The effect of past activities is limited only to the preceding one as we do not find such lagged effect extended to the second last activity (Appendix, Fig. S9).

Whilst the effect of LF/HF ratio during the current activity is robust to controlling for the duration of previous event (Table S5 specification (3)), the experienced physiological stress level of the previous event has no residual effect on the current positive feeling reported (specification (4)), despite the OR remaining below 1. The reported feeling from the last activity strongly correlates (positively) with current positive feeling (specification (5)) and can explain a significant proportion of its variation. Nevertheless, controlling for past positive feelings renders the effect of current LF/HF ratio insignificant (with *p*-value just below 10% level).

When we interrogate the effect of prolonged exposure to the last activity (Appendix, Fig. S10), we find that, apart from *relaxing*, activity duration does not moderate the effect of most of the activities on positive feelings reported for the next activity. Specifically, we find that an individual tends to feel less positive after an extended period of relaxation (*p* = 0.029), maybe due to the enjoyable positive feelings of relaxation that are relatively high and quite stable (Fig. 3) which may make a switch to another activity harder.

**Figure 5:**
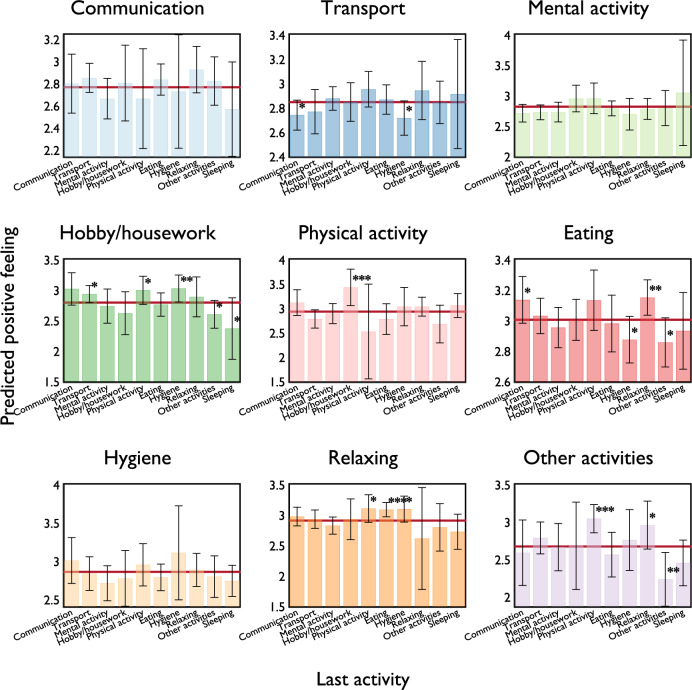
Effect of last activity on positive feeling. Each panel shows the predicted positive feeling of each activity combination. Red lines indicate the mean predicted positive feeling of each current activity. Error bars represent 95% CI of the predicted outcome. *, **, and *** indicate 10%, 5%, and 1% level of significance, respectively.

Next, we further examine how the preceding activities affect positive feelings reported for each type of activity conducted; for example, we can ask questions such as: do people feel more positive at work followed by a meal or some sleep? To assess these variations, we re-run the regression from specification (1) in Table S5 including the interaction terms between current and past activities. Naturally, certain sequences of activities are more common (e.g., *eating* preceding or followed by *mental activity*) while others are less common (e.g., hobby/housework and physical activity) (Appendix, Fig. S11). In Fig. 5, we show the predicted level of (current) positive feeling for each activity pair (e.g., *mental activity* following *eating*), with the solid red line indicating the predicted mean level of positive feeling about the current activity. Self-reported positive feelings for *communication, mental activity,* and *hygiene* do not seem to vary significantly due to prior activity. Individuals feel more positive during *hobby/housework* if past activities were *transport*, *physical activity*, and *hygiene*, while they are more negative after *sleeping.* For *physical activity*, past *hobby/housework* promotes positive feelings. For *eating*, past *hygiene* activity reduces positive feelings, while past *communication* or *relaxing* enhances positive feelings. Lastly, past *physical activity*, *eating*, and *hygiene* enhance the self-reported positive feeling during current *relaxation*.

## Discussion

The first goal of this analysis was to explore the association between subjective (self-reported) survey data and objective physiological measures. Such an analysis is interesting as technological innovations such as wearable biosensors that track those conditions throughout the day provide new ways of measuring physiological conditions. By using a large sample of repeated observations within a 24-h time frame (around 300 individuals with a total of 5000 activities), the study not only adds nuances to past research but allows a within-subject analysis that avoids the pitfalls and confounding factors that result from individual heterogeneity. We conclude that there is a robust positive association between self-reported psychological and objective physiological states as measured by HRV. Using a unique data set, the study corroborates previous claims that self-reported positive affect or hedonic well-being data are a reliable measure, which is good news for the social sciences in general given their reliance on survey-generated subjective data. As discussed, such self-reported data are frequently used in assessing the impacts of different types of health, psychological, or even educational interventions designed to affect individual well-being or positive affect. Perhaps more importantly for social scientists, it has even been suggested that public policy should be informed not only by GDP but also by bottom-up measures of self-reported well-being^40,41^. From such a policy perspective, our results imply that attempts by the OECD^8^ and other countries to measure positive affect (particularly, self-reported subjective well-being) as an indicator of a society’s progress or achievement and as a policy-making tool^62^ can meaningfully rely on self-reported data. At the same time, it is important to conduct more research that complements survey results with detailed high frequency data from biosensors, as these instruments can be worn constantly and offer the opportunity for truly longitudinal measurements.

With respect to activities, our results indicate that exercising, enjoying meals, or relaxing are associated with the strongest positive feelings. In terms of physiological stress, physical activities have the strongest positive effects. Beyond that we also find a positive spillover effect, namely that individuals report higher positive feelings during activities that follow a physical activity (relative to the baseline of mental activities). Such positive effects are also found for other activities such as eating and relaxing, as one would expect. We also find that individuals are more positive as the day progresses, perhaps due to feelings of achievement; although this effect may also be linked to biological adjustments throughout the day (e.g., drop in cortisol levels over the course of the day). However, when linking HRV directly to activities, it is clear that LF/HF ratio is lowest during activities in the early morning and highest in the afternoon. In fact, for most activities, LF/HF ratio increases as the day progresses and reaches its peak in the afternoon. For various activities such as eating, hygiene, communication, and hobby or housework, spending more time doing those activities is correlated with higher values of positive feelings. Yet, for less controllable activities such as travelling, the duration actually increases physiological stress. Such a result is consistent with the well-being literature, that does not use HRV methods, and shows more time spent commuting increases stress and lowers life satisfaction^63^, which raises the question of whether individuals are fully aware of the costs of travelling. In addition, other than our major finding that work does have an effect on positive feelings, most positive feeling results are consistent with the literature (for a comprehensive comparison see^64^). Communicating and sharing of emotional experiences are hedonically positive and contribute to the regulation of our emotions^65^; communicating with others makes us feel better; longer commute (transport) times increase stress and reduce positive feelings; commute times over 22 min have been found to reduce life satisfaction by as much as 0.103 points^63^. The mental activity result is stable, possibly because positive thinking makes us feel better while thinking negatively increases stress, anxiety, and depression^66^. Physical activity fosters normal growth and development and can make us feel better^67^; even housework in small amounts or engaging in a hobby helps^68^. Eating is associated with positive feelings^69^. Relaxing can make us feel better but laying around inactive for too long can induce negative feelings^64^. Duration itself may be linked to avoidance, procrastination, or even depression. Engaging in hygiene activities such as taking a long bath improves positive affect, especially in cultures like Japan^68^.

## Conclusion

In general, use of the HRV data helped us to better understand what happens as the duration of a single activity increases. Longer meetings and communication sessions increase our stress, as do longer commutes, too much mental activity, and too much exercise or physical activity. It is easy to understand why relaxing and taking longer over meals would induce little change in our stress levels (LF/HF ratio), as both are pleasurable activities, often shared with others and requiring little physical or mental effort. The duration of an activity can change how we feel, and affect our HRV, but in different directions.

Our results also provided insights into how prospection impacts positive affect. Emotions are not just about the past and the present. Emotions are intrinsically involved in extrapolating to the future and are not only in reaction to what happened before^70^. Activity protocols potentially provide a way of reducing conceptual and methodological errors when exploring the future by increasing, controllability, although we cannot identify what the individuals were thinking while doing those activities (e.g., whether or not and how they were mapping their possible futures). Nevertheless, our analyses show some consistent results; for example, individuals report higher positive feelings when their next task was to engage in physical activities, and it is better to relax after exercising, taking a bath, or enjoying a meal.

## Recommendations

We have provided evidence that the use of real time data collection provides a reliable real-time method for measuring feelings (positive affect). High cost medical heart rate monitors and the medical protocols used in this project may not be available to all researchers. However, the emergence of wearable devices that measure pulse and heart, HRV, stress, etc., open new research frontiers to all, and these primary source real-time data would be less likely to suffer from the biases inherent in survey data. The wearable devices like watches from Apple, Fitbit, Garmin, among others, dynamically collect pulse, heart rate and heart rate variability and other data that are accessible from their website (e.g.^71^). One could use the data from these inexpensive devices together with the data preparation and analysis methods offered in this paper to dynamically map in more detail individuals’ thinking process in relation to well-being and stress levels. In addition, the real-time intertemporal data provided by these devices allows us to pursue wellbeing-related questions that were previously constrained by data availability or survey recall and other biases; like how anticipation and adaption affects current mood, stress, or response to life event shocks, which can occur months or year(s) prior to well-being survey completion.

### Supplementary Information

Supplementary Information.

## Data Availability

The datasets generated and analysed during the current study are available in the Open Science Framework repository, https://osf.io/uq96m.
