# Supplementary material for: Positive affect and heart rate variability: a dynamic analysis
Source: Sci Rep. 2024 Mar 25;14:7004. doi: 10.1038/s41598-024-57279-5 (PMC10961327; doi:10.1038/s41598-024-57279-5)
Supplement: Supplementary file 1 — Supplementary Information. [file 41598_2024_57279_MOESM1_ESM.docx]

# Online Supplementary Material

**Analytical Model**

We use the STATA 17 command ‘feologit’ for the fixed-effects ordered logit model (Baetschmann, Staub & Winkelmann, 2015;) to explore within-subject covariation between psychological state (positive affect) and mental stress (HRV, log of LF/HF ratio) take the form:

$$y_{it}^{*}={\beta x}_{it}^{'}+\alpha_{i}+\varepsilon_{it}, i=1,\ldots,N t=1,\ldots,T. (1)$$

‘The fixed effects ordered logit model relates the latent variable *y** for individual *i* at time *t* to a linear index of observable characteristics *x_it_* and unobservable characteristics *α_i_*, and error term *ε_it_*’ (Baetschmann, Ballantyne, Staub & Winkelmann, 2020, p. 686-687).

This logit model helps overcome the conflict of opinion on the ordinal or cardinal nature of behavioral data collected using survey questions that are numerically scaled (e.g., 1, 2 ,3, 4, 5), where an individual’s, for example, ‘3’ may be different from another person’s ‘3’ or the gap between the numbers may vary. In addition, the fixed effect logit model provides for the inclusion of fixed effects which can account for time-invariant individual heterogeneity, like personal habits or personality, to reveal the true underlying dynamic effects.

**Extended Validation**

In assessing the dynamics of the LF/HF ratio, a key question arises: what drives the changes in the ratio – is it predominantly influenced by increased sympathetic (reflected in higher LF power) or decreased parasympathetic activity (lower HF power), or a combination of both? Understanding the relative contributions of LF and HF components to the LF/HF ratio may lead to a more nuanced understanding of the physiological responses to stress and the importance of sympathetic and parasympathetic control in maintaining autonomic balance. To this end, we employed a regression analysis to examine the log LF/HF ratio (as the dependent variable), regressing it against both LF and HF (log-transformed). Our approach involved standardizing LF, HF, and the LF/HF ratio within individual participants using a z-score method (by subtracting the mean and dividing by the standard deviation of each individual’s measures) and employing an individual fixed effects model, which enhance comparability across individuals by aligning the scales of these variables. The standardization approach also allows us to directly compare the influence of LF and HF on the ratio (beta coefficients, indicating the impact of a one-unit standard deviation change in LF or HF on the standardized LF/HF ratio).

The results suggest that HF has a relatively larger influence on the variation of the ratio (beta coefficient is -1.282; 95% CI = [-1.3, -1.26]) compared to LF (beta coefficient = 1.15; 95% CI = [1.13, 1.17]). The signs of these coefficients align with expectations, given the roles of HF and LF as the denominator and numerator in the ratio, respectively. Incorporating controls for variables like reported positive affect, types of activity, time of day, and activity duration in our analysis yielded results that were consistent with our initial findings. Additionally, when separately examining instances of increases and decreases in the LF/HF ratio, we consistently observed that changes in HF had a more pronounced impact on the LF/HF ratio (β = -1.25 and -1.22, respectively) compared to changes in LF (β = 1.09 and 1.14, respectively).

To corroborate these findings, we also conducted participant-specific regressions to assess the effects of HF and LF on the LF/HF ratio for each individual separately. The distribution of the coefficient estimates for HF and LF are visually represented in Figure S12a. Notably, these distributions are symmetric to each other and shared a highly negative correlation (ρ = -0.955). However, considering the absolute values of the HF coefficients (due to their negative impact on the LF/HF ratio) shows that the magnitude of (negative) effects is statistically larger for HF compared to LF (p < 0.001). Among our 320 participants, the mean estimated beta coefficient for LF is 1.90 (SD = 1.22), while the mean absolute value of HF beta coefficients is 2.05 (SD = 1.24), as illustrated in Figure S12b.

Furthermore, as discussed in section 3.2, it is important to acknowledge that a higher LF/HF ratio does not necessarily indicate change in overall HRV due to the cycle length dependence effect (changes in HR) and the continual adjustments of relative sympathovagal balance to meet regulatory system demands. We therefore show the relationship between LF/HF ratio and RMMSD (log), a time-domain measure of overall HRV (see Figure S12d). Expectedly, we see that the correlation between the two is negative (ρ = -0.263, p < 0.001), as RMSSD primarily reflects parasympathetic (vagal) activity with higher values indicate greater parasympathetic activity (more relaxed physiological state). Furthermore, we also show the regression results replicating Table S1 by replacing LF/HF ratio with RMSSD (see Table S6). The coefficient estimate of RMMSD are positive in all nine specifications, with six being statistically significant at 10% level. Thus, this result support those in our main analysis.

**Figure S1.** **Activity protocol.**

**
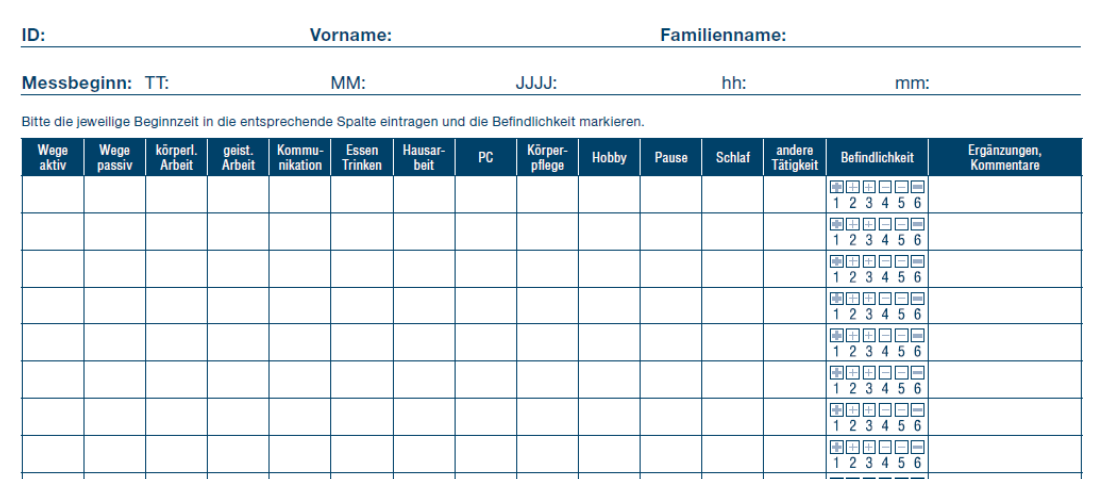
** Reported affect scale (Befindlichkeit). *ID*: Identification number, *Vorname*: first name, *Familienname*: last name, *Messbeginn*: start of the recording (day, month, year, hour, minutes). *Wege aktiv*: Active commute, *Wege passiv*: Passive commute, *Korperl. Arbeit*: Physical work, *geist. Arbeit*: Mental work, *Kommunikation*: Communication, *Essen Trinken*: Food/ beverages, *Hausarbeit*: Housework, *Hobby*: Hobby, *Pause*: Break, *Schlaf*: Sleep, *Andere Tatigkeit*: Other activity, *Befindlichkeit*: Mood (or mental state), *Erganzungen* *Kommentare*: Additional info/comments.

**Figure S2. Share of activities undertaken in 24-hour activity log.**

1,151 participants with 22,379 activities including sleep and activities with less than five minutes duration (**A**); 321 participants who completed mood assessment with 6,452 activities (**B**). Hourly activities are weighted by the activity duration per individual.

**Figure S3**. **Distribution of reported level of positive affect**.

Data from 5,412 (non-sleep) activities of 316 participants with mood assessment. The lowest two categories in the affect scale (see Figure S1) were merged into together (“0”), due to the skewness of its distribution.

**Figure S4**. **Distribution of positive affect across different activities.**

Data from 5,412 (non-sleep) activities of 316 participants with mood assessment.

**Figure S5**. **Noninvasive pocket-sized heart rate monitor**.


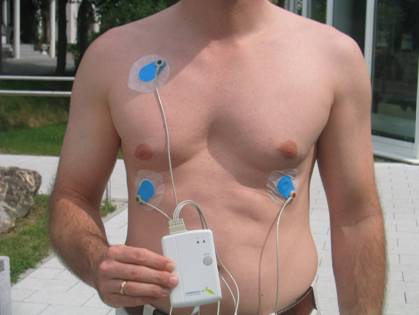


HRV was measured using the nonintrusive pocket-sized monitoring device (10x7x2.5cm) that connects to a human subject’s chest through three conducting electrodes. This device records the ECG (with a high sampling rate of 4096 Hz) and respiration levels.

**Figure S6**. **HRV distribution.**

Distribution of log LF/HF ratio values during 18,118 non-sleep activities from 1,151 participants (**A**); by participants with (*n* = 321) and without mood assessment (*n* = 831) (**B**).

**Figure S7. Age distribution**.

Age distribution of the 1,151 participants (**A**); by participants with (*n* = 321) and without mood assessment (*n* = 831) (**B**).

**Figure S8. Effect of types of activities and part of the day on mental stress (HRV).**

We show the coefficient estimates of types of activity and part of day from the fixed-effects regression of HRV (based on specification 3 from *Appendix*, Table S4). The reference categories are *mental activities* and *afternoon*, respectively. Error bars represent 95% CI. *, **, and *** indicate 10%, 5%, and 1% level of significance, respectively.

**Figure S9. Secondary effect from past activities.**

ORs obtained from fixed-effects ordered logit models of positive affect including indicators of last and second last activities. The reference category for activities is *mental activity*. Error bars represent 95% CI. *, **, and *** indicate 10%, 5%, and 1% level of significance, respectively.

**Figure S10. Duration of past activity on positive feeling.**

Predicted levels of positive affect (solid lines) are obtained from fixed-effects models with interaction terms between the type of past activity and its duration. Shaded areas represent 95% CI.

**Figure S11. Sequence of activities.**

Coloration shows the relative frequency of each pair of activities across all activity combinations (*N* = 5,280).

**Figure S12. Influence of LF and HF on LF/HF Ratio and Correlations with HR and RMSSD.**

Panel **a** displays the distribution of standardized beta coefficients derived from 320 regressions (one per participant, N = 320). Each regression models the log LF/HF ratio as the dependent variable with log LF and log HF as independent variables. All coefficients are statistically significant at the 1% level. Panel **b** presents the mean absolute values of the log LF and log HF coefficient estimates, accompanied by their 95% confidence intervals. Panels **c** and **d** illustrate the relationships between the log LF/HF ratio and two variables: heart rate (HR) and log RMMSD, respectively

**Table S1. Fixed-effects ordered logit models of positive affect**

|  | (1) | (2) | (3) | (4) | (5) | (6) | (7) | (8) | (9) |
| --- | --- | --- | --- | --- | --- | --- | --- | --- | --- |
| LF/HF ratio (log) | 0.849* | 0.812** | 0.808** | 0.803** | 0.806** | 0.804** | 0.810** | 0.802** | 0.810** |
|  | (0.079) | (0.075) | (0.076) | (0.075) | (0.076) | (0.076) | (0.074) | (0.076) | (0.075) |
| *Activity* |  |  |  |  |  |  |  |  |  |
| Communication |  | 1.137 | 1.110 | 1.128 | 1.117 | 1.144 | 1.020 | 1.121 | 0.971 |
|  |  | (0.128) | (0.124) | (0.127) | (0.124) | (0.131) | (0.183) | (0.126) | (0.152) |
| Transport |  | 1.165 | 1.146 | 1.174 | 1.166 | 1.193 | 1.521** | 1.198 | 1.236 |
|  |  | (0.131) | (0.131) | (0.135) | (0.136) | (0.139) | (0.310) | (0.142) | (0.207) |
| Hobby / Housework |  | 1.220 | 1.183 | 1.236 | 1.186 | 1.215 | 1.954*** | 1.200 | 0.979 |
|  |  | (0.193) | (0.186) | (0.195) | (0.191) | (0.194) | (0.508) | (0.190) | (0.213) |
| Physical activity |  | 1.490** | 1.513** | 1.510** | 1.503** | 1.523** | 1.229 | 1.520** | 1.396 |
|  |  | (0.245) | (0.247) | (0.251) | (0.245) | (0.252) | (0.307) | (0.248) | (0.300) |
| Eating |  | 2.000*** | 2.027*** | 2.066*** | 1.970*** | 2.072*** | 2.337*** | 2.106*** | 1.418** |
|  |  | (0.230) | (0.233) | (0.237) | (0.223) | (0.238) | (0.409) | (0.246) | (0.243) |
| Hygiene |  | 1.025 | 1.123 | 1.143 | 1.186 | 1.164 | 2.481* | 1.186 | 0.863 |
|  |  | (0.148) | (0.167) | (0.165) | (0.178) | (0.176) | (1.199) | (0.184) | (0.180) |
| Relaxing |  | 1.689*** | 1.653*** | 1.720*** | 1.695*** | 1.666*** | 1.648** | 1.706*** | 1.591*** |
|  |  | (0.231) | (0.222) | (0.229) | (0.233) | (0.223) | (0.338) | (0.233) | (0.277) |
| Other activity |  | 0.695** | 0.712* | 0.731* | 0.737* | 0.761 | 0.988 | 0.722* | 0.695 |
|  |  | (0.124) | (0.127) | (0.130) | (0.132) | (0.135) | (0.305) | (0.129) | (0.167) |
| *Part of the day* |  |  |  |  |  |  |  |  |  |
| Early morning |  |  | 0.664** | 0.739** |  |  | 0.983 | 0.667** | 0.672** |
|  |  |  | (0.111) | (0.104) |  |  | (0.547) | (0.112) | (0.113) |
| Morning |  |  | 1.010 | 0.991 |  |  | 1.196 | 1.017 | 1.021 |
|  |  |  | (0.104) | (0.106) |  |  | (0.203) | (0.104) | (0.104) |
| Night |  |  | 1.209* | 1.092 |  |  | 1.323 | 1.207* | 1.194 |
|  |  |  | (0.136) | (0.120) |  |  | (0.286) | (0.136) | (0.136) |
| Activity duration |  |  |  |  |  |  |  | 1.084* | 0.988 |
|  |  |  |  |  |  |  |  | (0.046) | (0.063) |
| Start hour FE | No | No | No | No | Yes | No | No | No | No |
| End hour FE | No | No | No | No | No | Yes | No | No | No |
| Activity*POD | No | No | No | No | No | No | Yes | No | No |
| Activity*Duration | No | No | No | No | No | No | No | No | Yes |
| Observations | 5414 | 5414 | 5414 | 5414 | 5414 | 5414 | 5414 | 5414 | 5414 |
| # individuals | 310 | 310 | 310 | 310 | 310 | 310 | 310 | 310 | 310 |
| Wald χ^2^ | 3.1 | 82.4 | 96.0 | 94.6 | 139.7 | 161.5 | 156.9 | 101.9 | 129.0 |
| Pseudo R^2^ | 0.001 | 0.015 | 0.019 | 0.017 | 0.026 | 0.025 | 0.025 | 0.020 | 0.023 |
| Prob > χ^2^ | 0.078 | 0.000 | 0.000 | 0.000 | 0.000 | 0.000 | 0.000 | 0.000 | 0.000 |

Notes: Dependent variable: Positive affect. Reported are odd ratios from the fixed-effects ordered logit model . Standard errors (clustered at individual level) in parentheses. Part of the day is defined by the start time of the activity in all specifications except for (4): early morning (12-7am); morning (7am-12pm); afternoon (12-5pm); and night (5pm-12am). In specification (4), we define part of the day using the end time of the activity with the same time cut-off. The symbols *, **, *** represent statistical significance at the 10%, 5% and 1% levels, respectively. Reference group: *Mental activity*; *afternoon*.

**Table S2. Fixed-effects ordered logit models using BUC-τ estimator**

|  | (1) | (2) | (3) | (4) | (5) | (6) | (7) | (8) | (9) |
| --- | --- | --- | --- | --- | --- | --- | --- | --- | --- |
| LF/HF ratio (log) | 0.888 | 0.851* | 0.848* | 0.840* | 0.845* | 0.838* | 0.847* | 0.842* | 0.849* |
|  | (0.081) | (0.077) | (0.078) | (0.077) | (0.078) | (0.077) | (0.077) | (0.078) | (0.078) |
| *Activity* |  |  |  |  |  |  |  |  |  |
| Communication |  | 1.121 | 1.094 | 1.114 | 1.089 | 1.128 | 0.970 | 1.106 | 0.975 |
|  |  | (0.133) | (0.130) | (0.133) | (0.129) | (0.136) | (0.175) | (0.132) | (0.161) |
| Transport |  | 1.128 | 1.109 | 1.141 | 1.120 | 1.153 | 1.451* | 1.158 | 1.234 |
|  |  | (0.123) | (0.124) | (0.128) | (0.127) | (0.131) | (0.285) | (0.133) | (0.197) |
| Hobby / Housework |  | 1.178 | 1.142 | 1.197 | 1.136 | 1.178 | 1.796** | 1.161 | 0.959 |
|  |  | (0.176) | (0.171) | (0.179) | (0.173) | (0.179) | (0.454) | (0.174) | (0.199) |
| Physical activity |  | 1.448** | 1.478** | 1.478** | 1.465** | 1.496** | 1.245 | 1.485** | 1.429* |
|  |  | (0.233) | (0.237) | (0.240) | (0.234) | (0.241) | (0.303) | (0.238) | (0.293) |
| Eating |  | 1.954*** | 1.976*** | 2.025*** | 1.897*** | 2.025*** | 2.397*** | 2.051*** | 1.481** |
|  |  | (0.218) | (0.219) | (0.226) | (0.209) | (0.226) | (0.412) | (0.232) | (0.240) |
| Hygiene |  | 1.050 | 1.148 | 1.179 | 1.206 | 1.197 | 2.449 | 1.209 | 0.947 |
|  |  | (0.149) | (0.166) | (0.169) | (0.179) | (0.180) | (1.341) | (0.181) | (0.195) |
| Relaxing |  | 1.634*** | 1.590*** | 1.664*** | 1.618*** | 1.621*** | 1.711*** | 1.640*** | 1.628*** |
|  |  | (0.216) | (0.206) | (0.216) | (0.217) | (0.214) | (0.351) | (0.217) | (0.278) |
| Other activity |  | 0.717* | 0.734* | 0.757 | 0.746* | 0.785 | 1.051 | 0.743* | 0.750 |
|  |  | (0.124) | (0.128) | (0.132) | (0.131) | (0.137) | (0.299) | (0.130) | (0.173) |
| *Part of the day* |  |  |  |  |  |  |  |  |  |
| Early morning |  |  | 0.669** | 0.730** |  |  | 0.887 | 0.673** | 0.676** |
|  |  |  | (0.108) | (0.100) |  |  | (0.469) | (0.109) | (0.109) |
| Morning |  |  | 1.014 | 0.980 |  |  | 1.188 | 1.020 | 1.023 |
|  |  |  | (0.102) | (0.103) |  |  | (0.208) | (0.103) | (0.103) |
| Night |  |  | 1.224* | 1.102 |  |  | 1.418 | 1.222* | 1.216* |
|  |  |  | (0.132) | (0.117) |  |  | (0.303) | (0.132) | (0.133) |
| Activity duration |  |  |  |  |  |  |  | 1.077* | 1.011 |
|  |  |  |  |  |  |  |  | (0.042) | (0.062) |
|  |  |  |  |  |  |  |  |  | (0.127) |
| Start hour FE | No | No | No | No | Yes | No | No | No | No |
| End hour FE | No | No | No | No | No | Yes | No | No | No |
| Activity*POD | No | No | No | No | No | No | Yes | No | No |
| Activity*Duration | No | No | No | No | No | No | No | No | Yes |
| Observations | 5461 | 5461 | 5461 | 5461 | 5461 | 5461 | 5461 | 5461 | 5461 |
| # individuals | 315 | 315 | 315 | 315 | 315 | 315 | 315 | 315 | 315 |
| Wald χ^2^ | 992.3 | 1056.9 | 1052.2 | 1102.4 | 1351.2 | 1382.6 | 1435.7 | 1054.0 | 1114.3 |
| Pseudo R^2^ | 0.412 | 0.420 | 0.423 | 0.422 | 0.427 | 0.425 | 0.426 | 0.423 | 0.425 |
| Prob > χ^2^ | 0.000 | 0.000 | 0.000 | 0.000 | 0.000 | 0.000 | 0.000 | 0.000 | 0.000 |

Notes: Dependent variable: Positive affect. Reported are odd ratios from the fixed-effects ordered logit model . Standard errors (clustered at individual level) in parentheses. Part of the day is defined by the start time of the activity in all specifications except for (4): early morning (12-7am); morning (7am-12pm); afternoon (12-5pm); and night (5pm-12am). In specification (4), we define part of the day using the end time of the activity with the same time cut-off. We employ the BUC-τ estimator (Baetschmann, 2012) which requires additional assumption of constant thresholds across individuals to the underlying ordered logit model. Using the generalized Hausman specification test, we find no statistically significant difference between the results from the blowup and cluster (BUC) and BUC-τ estimators. Both estimators are thus appropriate and offer consistent estimates of the slope parameters. The symbols *, **, *** represent statistical significance at the 10%, 5% and 1% levels, respectively. Reference group: *Mental activity*; *afternoon*.

**Table S3. Linear panel fixed-effects model**

|  | (1) | (2) | (3) | (4) | (5) | (6) | (7) | (8) | (9) |
| --- | --- | --- | --- | --- | --- | --- | --- | --- | --- |
| LF/HF ratio (log) | -0.060* | -0.074** | -0.076** | -0.079** | -0.078** | -0.079** | -0.077** | -0.079** | -0.076** |
|  | (0.034) | (0.034) | (0.034) | (0.034) | (0.034) | (0.034) | (0.034) | (0.034) | (0.034) |
| *Activity* |  |  |  |  |  |  |  |  |  |
| Communication |  | 0.045 | 0.037 | 0.043 | 0.036 | 0.048 | 0.001 | 0.042 | -0.008 |
|  |  | (0.042) | (0.042) | (0.043) | (0.042) | (0.043) | (0.067) | (0.042) | (0.060) |
| Transport |  | 0.055 | 0.050 | 0.059 | 0.054 | 0.063 | 0.144** | 0.067 | 0.082 |
|  |  | (0.041) | (0.041) | (0.041) | (0.042) | (0.042) | (0.071) | (0.042) | (0.059) |
| Hobby / Housework |  | 0.073 | 0.063 | 0.079 | 0.065 | 0.072 | 0.231*** | 0.070 | -0.001 |
|  |  | (0.056) | (0.055) | (0.055) | (0.057) | (0.056) | (0.089) | (0.056) | (0.078) |
| Physical activity |  | 0.136** | 0.142** | 0.144** | 0.140** | 0.148*** | 0.063 | 0.145** | 0.116 |
|  |  | (0.057) | (0.056) | (0.057) | (0.056) | (0.057) | (0.096) | (0.056) | (0.074) |
| Eating |  | 0.235*** | 0.239*** | 0.247*** | 0.227*** | 0.246*** | 0.296*** | 0.253*** | 0.159*** |
|  |  | (0.040) | (0.039) | (0.040) | (0.039) | (0.040) | (0.062) | (0.040) | (0.056) |
| Hygiene |  | 0.012 | 0.044 | 0.051 | 0.062 | 0.057 | 0.345* | 0.064 | -0.004 |
|  |  | (0.052) | (0.053) | (0.051) | (0.053) | (0.054) | (0.177) | (0.055) | (0.069) |
| Relaxing |  | 0.180*** | 0.171*** | 0.187*** | 0.174*** | 0.174*** | 0.175** | 0.183*** | 0.173*** |
|  |  | (0.047) | (0.046) | (0.046) | (0.047) | (0.046) | (0.068) | (0.047) | (0.060) |
| Other activity |  | -0.132** | -0.123* | -0.115* | -0.115* | -0.098 | 0.001 | -0.117* | -0.126 |
|  |  | (0.067) | (0.067) | (0.066) | (0.067) | (0.066) | (0.109) | (0.067) | (0.091) |
| *Part of the day* |  |  |  |  |  |  |  |  |  |
| Early morning |  |  | -0.152** | -0.113** |  |  | -0.017 | -0.150** | -0.147** |
|  |  |  | (0.061) | (0.051) |  |  | (0.214) | (0.062) | (0.061) |
| Morning |  |  | 0.002 | -0.006 |  |  | 0.062 | 0.004 | 0.005 |
|  |  |  | (0.036) | (0.038) |  |  | (0.063) | (0.036) | (0.036) |
| Night |  |  | 0.065* | 0.030 |  |  | 0.100 | 0.063 | 0.061 |
|  |  |  | (0.039) | (0.038) |  |  | (0.077) | (0.039) | (0.039) |
| Activity duration |  |  |  |  |  |  |  | 0.026* | -0.000 |
|  |  |  |  |  |  |  |  | (0.014) | (0.021) |
| Start hour FE | No | No | No | No | Yes | No | No | No | No |
| End hour FE | No | No | No | No | No | Yes | No | No | No |
| Activity*POD | No | No | No | No | No | No | Yes | No | No |
| Activity*Duration | No | No | No | No | No | No | No | No | Yes |
| Observations | 5499 | 5499 | 5499 | 5499 | 5499 | 5499 | 5499 | 5499 | 5499 |
| Number of individuals | 320 | 320 | 320 | 320 | 320 | 320 | 320 | 320 | 320 |
| R-square | 0.000 | 0.011 | 0.015 | 0.013 | 0.019 | 0.017 | 0.017 | 0.015 | 0.017 |
| Prob. > F | 0.081 | 0.000 | 0.000 | 0.000 | 0.000 | 0.000 | 0.000 | 0.000 | 0.000 |

Notes: Dependent variable: Positive affect. Standard errors (clustered at individual level) in parentheses. Part of the day is defined by the start time of the activity in all specifications except for (4): early morning (12-7am); morning (7am-12pm); afternoon (12-5pm); and night (5pm-12am). In specification (4), we define part of the day using the end time of the activity with the same time cut-off. The symbols *, **, *** represent statistical significance at the 10%, 5% and 1% levels, respectively. Reference group: *Mental activity*; *afternoon*.

**Table S4. Panel fixed-effects linear model of HRV**

|  | (1) | (2) | (3) | (4) | (5) | (6) | (7) | (8) |
| --- | --- | --- | --- | --- | --- | --- | --- | --- |
| *Activity* |  |  |  |  |  |  |  |  |
| Communication | 0.034* | 0.038** | 0.037* | 0.039** | 0.039** | -0.005 | 0.041** | 0.036 |
|  | (0.019) | (0.019) | (0.019) | (0.019) | (0.019) | (0.028) | (0.019) | (0.027) |
| Transport | -0.186*** | -0.175*** | -0.174*** | -0.169*** | -0.169*** | -0.193*** | -0.165*** | -0.198*** |
|  | (0.019) | (0.019) | (0.019) | (0.019) | (0.019) | (0.030) | (0.019) | (0.028) |
| Hobby/ housework | -0.144*** | -0.122*** | -0.123*** | -0.113*** | -0.113*** | -0.161*** | -0.117*** | -0.110*** |
|  | (0.023) | (0.022) | (0.022) | (0.023) | (0.022) | (0.033) | (0.023) | (0.034) |
| Physical activity | -0.347*** | -0.336*** | -0.335*** | -0.327*** | -0.329*** | -0.276*** | -0.333*** | -0.340*** |
|  | (0.040) | (0.040) | (0.040) | (0.039) | (0.039) | (0.063) | (0.040) | (0.058) |
| Eating | 0.003 | 0.016 | 0.015 | 0.023 | 0.023 | 0.023 | 0.025 | 0.045 |
|  | (0.018) | (0.018) | (0.018) | (0.019) | (0.019) | (0.027) | (0.019) | (0.028) |
| Hygiene | -0.201*** | -0.160*** | -0.159*** | -0.145*** | -0.147*** | -0.097 | -0.147*** | -0.184*** |
|  | (0.025) | (0.025) | (0.025) | (0.026) | (0.026) | (0.095) | (0.025) | (0.034) |
| Relaxing | -0.011 | 0.003 | 0.002 | 0.006 | 0.006 | 0.021 | 0.010 | 0.025 |
|  | (0.023) | (0.023) | (0.022) | (0.023) | (0.022) | (0.033) | (0.023) | (0.032) |
| Other activities | -0.118*** | -0.093*** | -0.093*** | -0.091*** | -0.091*** | -0.189*** | -0.089*** | -0.115*** |
|  | (0.024) | (0.024) | (0.024) | (0.025) | (0.024) | (0.043) | (0.024) | (0.032) |
| *Part of the day* |  |  |  |  |  |  |  |  |
| Early morning |  | -0.130*** | -0.119*** |  |  | -0.196** | -0.128*** | -0.127*** |
|  |  | (0.031) | (0.024) |  |  | (0.082) | (0.031) | (0.031) |
| Morning |  | -0.059*** | -0.059*** |  |  | -0.047 | -0.057*** | -0.056*** |
|  |  | (0.017) | (0.018) |  |  | (0.030) | (0.017) | (0.017) |
| Night |  | -0.071*** | -0.063*** |  |  | -0.126*** | -0.072*** | -0.070*** |
|  |  | (0.017) | (0.016) |  |  | (0.031) | (0.017) | (0.017) |
| Activity duration |  |  |  |  |  |  | 0.016** | 0.013 |
|  |  |  |  |  |  |  | (0.006) | (0.010) |
| Start hour FE | No | No | No | Yes | No | No | No | No |
| End hour FE | No | No | No | No | Yes | No | No | No |
| Activity*POD | No | No | No | No | No | Yes | No | Yes |
| Observations | 5574 | 5574 | 5574 | 5574 | 5574 | 5574 | 5574 | 5574 |
| # individuals | 321 | 321 | 321 | 321 | 321 | 321 | 321 | 321 |
| R-square | 0.050 | 0.053 | 0.053 | 0.054 | 0.055 | 0.058 | 0.052 | 0.052 |
| Prob. > F | 0.000 | 0.000 | 0.000 | 0.000 | 0.000 | 0.000 | 0.000 | 0.000 |

Notes: Dependent variable: LF/HF ratio (log). Standard errors (clustered at individual level) in parentheses. Part of the day is defined by the start time of the activity in all specifications except for (4): early morning (12-7am); morning (7am-12pm); afternoon (12-5pm); and night (5pm-12am). In specification (4), we define part of the day using the end time of the activity with the same time cut-off. The symbols *, **, *** represent statistical significance at the 10%, 5% and 1% levels, respectively. Reference group: *Mental activity*; *afternoon.*

**Table S5. Fixed-effects ordered logit models with previous and next activities**

|  | (1) | (2) | (3) | (4) | (5) |
| --- | --- | --- | --- | --- | --- |
| LF/HF ratio (log) | 0.814** | 0.796** | 0.814** | 0.829** | 0.866 |
|  | (0.080) | (0.078) | (0.080) | (0.078) | (0.076) |
| *Current activity* |  |  |  |  |  |
| Communication | 1.080 | 1.004 | 1.082 | 1.074 | 1.074 |
|  | (0.143) | (0.132) | (0.143) | (0.141) | (0.138) |
| Transport | 1.194 | 1.109 | 1.201 | 1.196 | 1.217 |
|  | (0.152) | (0.140) | (0.154) | (0.154) | (0.154) |
| Hobby/housework | 1.198 | 1.082 | 1.206 | 1.193 | 1.189 |
|  | (0.201) | (0.179) | (0.203) | (0.201) | (0.195) |
| Physical activity | 1.591*** | 1.474** | 1.589*** | 1.596*** | 1.468** |
|  | (0.284) | (0.257) | (0.284) | (0.285) | (0.266) |
| Eating | 2.141*** | 2.036*** | 2.162*** | 2.156*** | 2.196*** |
|  | (0.266) | (0.258) | (0.269) | (0.268) | (0.266) |
| Hygiene | 1.234 | 1.094 | 1.255 | 1.250 | 1.205 |
|  | (0.197) | (0.177) | (0.201) | (0.200) | (0.195) |
| Relaxing | 1.722*** | 1.515*** | 1.723*** | 1.713*** | 1.809*** |
|  | (0.247) | (0.231) | (0.247) | (0.246) | (0.242) |
| Other activities | 0.736 | 0.646** | 0.743 | 0.736 | 0.778 |
|  | (0.141) | (0.121) | (0.142) | (0.141) | (0.147) |
| *Part of the day* |  |  |  |  |  |
| Early morning | 0.766 | 0.660** | 0.783 | 0.777 | 0.832 |
|  | (0.137) | (0.116) | (0.142) | (0.140) | (0.140) |
| Morning | 1.065 | 1.029 | 1.074 | 1.068 | 1.152 |
|  | (0.115) | (0.112) | (0.117) | (0.116) | (0.107) |
| Night | 1.196 | 1.249* | 1.203 | 1.200 | 1.209* |
|  | (0.145) | (0.150) | (0.147) | (0.146) | (0.122) |
| Activity duration | 1.096** | 1.090** | 1.092** | 1.091** | 1.100** |
|  | (0.047) | (0.047) | (0.047) | (0.047) | (0.048) |
| *Last/next activity* |  |  |  |  |  |
| Communication | 1.163 | 0.954 | 1.158 | 1.160 | 1.124 |
|  | (0.144) | (0.124) | (0.144) | (0.144) | (0.141) |
| Transport | 1.165 | 0.999 | 1.144 | 1.125 | 1.065 |
|  | (0.144) | (0.114) | (0.142) | (0.141) | (0.125) |
| Hobby/housework | 1.288 | 1.005 | 1.274 | 1.253 | 1.182 |
|  | (0.218) | (0.139) | (0.216) | (0.210) | (0.199) |
| Physical activity | 1.611** | 1.344* | 1.603** | 1.557** | 1.341 |
|  | (0.301) | (0.219) | (0.299) | (0.286) | (0.251) |
| Eating | 1.234* | 1.151 | 1.215* | 1.221* | 1.008 |
|  | (0.143) | (0.136) | (0.141) | (0.143) | (0.123) |
| Hygiene | 1.118 | 0.943 | 1.084 | 1.069 | 0.991 |
|  | (0.172) | (0.151) | (0.167) | (0.164) | (0.155) |
| Relaxing | 1.497*** | 0.859 | 1.472** | 1.472** | 1.306* |
|  | (0.234) | (0.116) | (0.231) | (0.231) | (0.211) |
| Other activities | 0.842 | 0.745* | 0.836 | 0.827 | 0.942 |
|  | (0.138) | (0.123) | (0.137) | (0.135) | (0.157) |
| Sleeping | 0.796 | 1.070 | 0.899 | 0.893 | 0.831 |
|  | (0.132) | (0.164) | (0.199) | (0.197) | (0.196) |
| Activity duration_j-1_ |  |  | 0.969 | 0.969 | 0.965 |
|  |  |  | (0.032) | (0.032) | (0.034) |
| LF/HF ratio (log)_j-1_ |  |  |  | 0.913 | 0.960 |
|  |  |  |  | (0.082) | (0.090) |
| Positive feeling_j-1_ |  |  |  |  | 1.826*** |
|  |  |  |  |  | (0.092) |
| Activity set | Last | Next | Last | Last | Last |
| Observations | 5058 | 5057 | 5058 | 5057 | 5040 |
| Number of individuals | 305 | 305 | 305 | 305 | 304 |
| Wald χ^2^ | 117.337 | 113.041 | 118.806 | 120.937 | 304.424 |
| Pseudo R^2^ | 0.025 | 0.023 | 0.025 | 0.026 | 0.081 |
| Prob > χ^2^ | 0.000 | 0.000 | 0.000 | 0.000 | 0.000 |

Notes: Standard errors (clustered at individual level) in parentheses. The symbols *, **, *** represent statistical significance at the 10%, 5% and 1% levels, respectively.

**Table S6. Fixed-effects ordered logit models of positive affect with RMMSD**

|  | (1) | (2) | (3) | (4) | (5) | (6) | (7) | (8) | (9) |
| --- | --- | --- | --- | --- | --- | --- | --- | --- | --- |
| RMMSD (log) | 1.041 | 1.068 | 1.077* | 1.074* | 1.079* | 1.083* | 1.081* | 1.073* | 1.065 |
|  | (0.037) | (0.043) | (0.043) | (0.043) | (0.045) | (0.045) | (0.043) | (0.043) | (0.043) |
| *Activity* |  |  |  |  |  |  |  |  |  |
| Communication |  | 1.113 | 1.083 | 1.101 | 1.090 | 1.116 | 1.003 | 1.093 | 0.949 |
|  |  | (0.126) | (0.121) | (0.125) | (0.122) | (0.129) | (0.180) | (0.123) | (0.149) |
| Transport |  | 1.185 | 1.162 | 1.193 | 1.184 | 1.210 | 1.546** | 1.212 | 1.267 |
|  |  | (0.137) | (0.137) | (0.141) | (0.140) | (0.145) | (0.317) | (0.148) | (0.218) |
| Hobby / Housework |  | 1.215 | 1.168 | 1.225 | 1.172 | 1.199 | 1.926** | 1.187 | 0.978 |
|  |  | (0.198) | (0.190) | (0.199) | (0.193) | (0.197) | (0.503) | (0.194) | (0.216) |
| Physical activity |  | 1.522** | 1.537*** | 1.539** | 1.531*** | 1.547*** | 1.233 | 1.550*** | 1.433* |
|  |  | (0.255) | (0.256) | (0.260) | (0.253) | (0.260) | (0.307) | (0.258) | (0.313) |
| Eating |  | 1.979*** | 2.000*** | 2.040*** | 1.947*** | 2.049*** | 2.301*** | 2.071*** | 1.395* |
|  |  | (0.228) | (0.229) | (0.234) | (0.220) | (0.235) | (0.401) | (0.242) | (0.239) |
| Hygiene |  | 0.987 | 1.068 | 1.091 | 1.124 | 1.099 | 2.402* | 1.127 | 0.848 |
|  |  | (0.157) | (0.172) | (0.171) | (0.181) | (0.180) | (1.131) | (0.190) | (0.184) |
| Relaxing |  | 1.682*** | 1.638*** | 1.708*** | 1.678*** | 1.646*** | 1.618** | 1.687*** | 1.575*** |
|  |  | (0.232) | (0.221) | (0.229) | (0.232) | (0.223) | (0.333) | (0.232) | (0.277) |
| Other activity |  | 0.695** | 0.708* | 0.728* | 0.733* | 0.756 | 0.979 | 0.717* | 0.690 |
|  |  | (0.124) | (0.127) | (0.130) | (0.131) | (0.134) | (0.302) | (0.129) | (0.167) |
| *Part of the day* |  |  |  |  |  |  |  |  |  |
| Early morning |  |  | 0.671** |  |  |  | 1.000 | 0.675** | 0.680** |
|  |  |  | (0.113) |  |  |  | (0.565) | (0.115) | (0.115) |
| Morning |  |  | 1.011 |  |  |  | 1.194 | 1.018 | 1.022 |
|  |  |  | (0.104) |  |  |  | (0.202) | (0.104) | (0.104) |
| Night |  |  | 1.224* |  |  |  | 1.345 | 1.222* | 1.208* |
|  |  |  | (0.136) |  |  |  | (0.291) | (0.136) | (0.136) |
| Activity duration |  |  |  |  |  |  |  | 1.076* | 0.983 |
|  |  |  |  |  |  |  |  | (0.045) | (0.063) |
| Start hour FE | No | No | No | No | Yes | No | No | No | No |
| End hour FE | No | No | No | No | No | Yes | No | No | No |
| Activity*POD | No | No | No | No | No | No | Yes | No | No |
| Activity*Duration | No | No | No | No | No | No | No | No | Yes |
| Observations | 5415 | 5415 | 5415 | 5415 | 5415 | 5415 | 5415 | 5415 | 5415 |
| # individuals | 310 | 310 | 310 | 310 | 310 | 310 | 310 | 310 | 310 |
| Wald χ^2^ | 1.3 | 79.6 | 90.9 | 89.6 | 132.1 | 151.1 | 157.2 | 95.9 | 123.9 |
| Pseudo R^2^ | 0.000 | 0.014 | 0.019 | 0.017 | 0.025 | 0.024 | 0.024 | 0.019 | 0.023 |
| Prob > χ^2^ | 0.261 | 0.000 | 0.000 | 0.000 | 0.000 | 0.000 | 0.000 | 0.000 | 0.000 |

Notes: Dependent variable: Positive affect. Reported are odd ratios from the fixed-effects ordered logit model. Standard errors (clustered at individual level) in parentheses. Part of the day is defined by the start time of the activity in all specifications except for (4): early morning (12-7am); morning (7am-12pm); afternoon (12-5pm); and night (5pm-12am). In specification (4), we define part of the day using the end time of the activity with the same time cut-off. The symbols *, **, *** represent statistical significance at the 10%, 5% and 1% levels, respectively. Reference group: *Mental activity*; *afternoon*.
